# From “Kidneys Govern Bones” to Chronic Kidney Disease, Diabetes Mellitus, and Metabolic Bone Disorder: A Crosstalk between Traditional Chinese Medicine and Modern Science

**DOI:** 10.1155/2016/4370263

**Published:** 2016-09-07

**Authors:** Xiao-Qin Wang, Xin-Rong Zou, Yuan Clare Zhang

**Affiliations:** ^1^Department of Chinese Medicine Nephrology, Hubei Provincial Hospital of TCM, Hubei University of Chinese Medicine, Wuhan, Hubei Province, China; ^2^Y. Clare Zhang Practice of Oriental Medicine, Tucson, AZ, USA

## Abstract

Although traditional Chinese medicine (TCM) and Western medicine have evolved on distinct philosophical foundations and reasoning methods, an increasing body of scientific data has begun to reveal commonalities. Emerging scientific evidence has confirmed the validity and identified the molecular mechanisms of many ancient TCM theories. One example is the concept of “Kidneys Govern Bones.” Here we discuss the molecular mechanisms supporting this theory and its potential significance in treating complications of chronic kidney disease (CKD) and diabetes mellitus. Two signaling pathways essential for calcium-phosphate metabolism can mediate the effect of kidneys in bone homeostasis, one requiring renal production of bioactive vitamin D and the other involving an endocrine axis based on kidney-expressed Klotho and bone-secreted fibroblast growth factor 23. Disruption of either pathway can lead to calcium-phosphate imbalance and vascular calcification, accelerating metabolic bone disorder. Chinese herbal medicine is an adjunct therapy widely used for treating CKD and diabetes. Our results demonstrate the therapeutic effects and underlying mechanisms of a Chinese herbal formulation, Shen-An extracts, in diabetic nephropathy and renal osteodystrophy. We believe that the smart combination of Eastern and Western concepts holds great promise for inspiring new ideas and therapies for preventing and treating complications of CKD and diabetes.

## 1. “Kidneys Govern Bones” Theory in Traditional Chinese Medicine

In traditional Chinese medicine (TCM), bone growth and development are interrelated with kidney function. This concept first appeared in the* Yellow Emperor's Inner Classic*. This book was written before 100 BC but is still considered the most important pillar of TCM because it established the fundamental TCM theories that remain highly relevant and inspire new therapeutic ideas today. The first chapter of the Inner Classic [1] pointed out the intrinsic connection between the natural progression of human life (in terms of growth, development, reproduction, and aging) and changes in bone conditions and that both are controlled by Kidney Essence (i.e., Kidney Jing). This is the origin of the theory known as “Kidneys Store Essence and Govern Bones.” In short, we call it “Kidneys Govern Bones.”

The meaning of “Kidneys Govern Bones” is twofold [2]. In a broad sense, kidneys store Essence. Kidney Essence is composed of Pre-Heaven Essence and Post-Heaven Essence. Pre-Heaven Essence refers to the genetic factors and constitutional conditions inherited from the parents prior to birth. Post-Heaven Essence refers to the nutritional nourishment received from foods and the physiological reserve/surplus generated by metabolic functions of the body following birth. Kidney Essence is the critical substance and foundation underlying the entire human life, including growth, development, reproduction, aging, and all functions. Kidney Essence generates Kidney Qi, which turns into Kidney Yin and Kidney Yang. Kidney Yang promotes and drives growth, and Kidney Yin moistens and nourishes organs and tissues. They are intercontrolling and interdependent. Delicate interaction between the two ensures that the body remains in an ever changing yet instantly balanced state. Deficiency of Kidney Essence or disturbance in the Yin-Yang balance can influence progression of chronic diseases and accelerate the aging process. On the other hand, in a narrow sense Kidney Essence is critical to skeletal development. Kidney Essence in its abundance can nourish bone marrow and strengthen bone structure. Kidney Essence Deficiency can contribute to late closing of fontanel and osteomalacia in children and osteoporosis and increased fracture risk in the elderly.

Based on this theory, generations of TCM doctors have emphasized tonifying Kidney Essence for the treatment of various bone disorders and chronic diseases and have also used this strategy to slow down aging processes. This approach has consistently proven effective in clinical practice. For instance, Gu Sui Bu (*Drynaria fortunei*), a kidney-tonifying herb, can promote osteoblast differentiation and maturation and improve bone mass and strength [3, 4]. Herbs that tonify Kidney Yang, for example, Xian Mao (*Curculigo orchioides*), Yin Yang Huo (*Herba Epimedii*), Ba Ji Tian (*Morinda officinalis*), Rou Cong Rong (*Cistanche deserticola*), Du Zhong (*Eucommia ulmoides*), Bu Gu Zhi (*Psoralea corylifolia*), Huang Qi (*Astragalus membranaceus*), Tu Si Zi (*Cuscuta chinensis*), and Xu Duan (*Dipsacus asper*), and herbs that tonify Kidney Yin, for example, Nu Zhen Zi (*Fructus Ligustri Lucidi*), offer significant protection for age-related and drug-induced osteoporosis [5–7]. Liao et al. surveyed 115,327 patients in Taiwan who had newly diagnosed fractures [7]. Five percent of these patients used Chinese herbal medicine adjunctively for fracture treatment. These patients had significantly faster recovery and lower medical expenses during the six months following fracture, compared to nonherb users. Interestingly, the incidences of cardiovascular complications, chronic obstructive pulmonary disease, diabetes mellitus, and stroke were also significantly lower in the herb-treated patients.

## 2. Molecular Mechanisms of “Kidneys Govern Bones”: Vitamin D and FGF23-Klotho

“Kidneys Govern Bones” is an ancient theory that has sustained the test of time in clinical practice of Chinese medicine. However, how to understand this theory in the context of the biochemical and molecular mechanisms of modern science has been an intriguing question.

The successful treatment of rickets in children and osteomalacia in adults using vitamin D illustrates an important role that the kidneys play in bone homeostasis. Vitamin D_3_ is synthesized in the epidermis in response to UV-B, converted to 25(OH)D_3_ in the liver, and then transported to the kidneys where it is converted to the biologically active vitamin D, 1,25(OH)_2_D_3_, also known as calcitriol. Calcitriol and parathyroid hormone (PTH) provide tight control of plasma calcium levels through a negative feedback loop that regulates three pathways: intestinal uptake, renal reabsorption, and skeletal release of calcium and phosphate. Calcitriol can elevate plasma levels of calcium and phosphate, resulting in mineralization of the skeleton and greater bone density (reviewed in [8]). In Chinese medicine, Kidney Qi Deficiency, or poor kidney function, leads to pathologically low calcitriol levels, leading to osteoporosis and osteomalacia. Therefore, vitamin D_3_ is less effective for treating bone diseases caused by chronic kidney failure, as it cannot be rendered active by the kidneys. For these patients, calcitriol or its active analogues are a better treatment choice [9, 10].

Discoveries since 2000 have identified a new bone-kidney endocrine axis, providing further insight into the molecular mechanisms underlying the theory of “Kidneys Govern Bones.” This bone-kidney axis, based on the bone-secreted FGF23 and kidney-expressed Klotho, plays a crucial role in calcium-phosphate metabolism and vitamin D regulation [11].

Fibroblast growth factors (FGFs) are a diverse family of intracellular, autocrine/paracrine, and endocrine factors that regulate cell growth, transformation, and metabolism. FGF23, a member of the FGF19 subfamily of endocrine FGFs, is mainly secreted by osteoblasts and osteocytes [12, 13]. Endocrine FGFs require cofactors for the stable binding to FGF receptors (FGFRs) and subsequent local signaling. Cofactor expression profiles determine the signaling specificity of endocrine FGFs [14].

One FGF cofactor is Klotho, an “antiaging” protein discovered in 1997 [15, 16]. Klotho is a transmembrane protein and cofactor for FGF23 and is important in calcium-phosphate homeostasis. Klotho is predominantly expressed in the kidneys, the choroid plexus of the brain, and the parathyroid gland [17]. Klotho expressed in the distal convoluted tubules of the kidneys binds to FGF receptor 1c (FGFR1c), which converts FGFR1c to an FGF23-specific receptor [18]. The kidneys therefore become a target organ for FGF23 secreted by bones.

**Figure 1:**
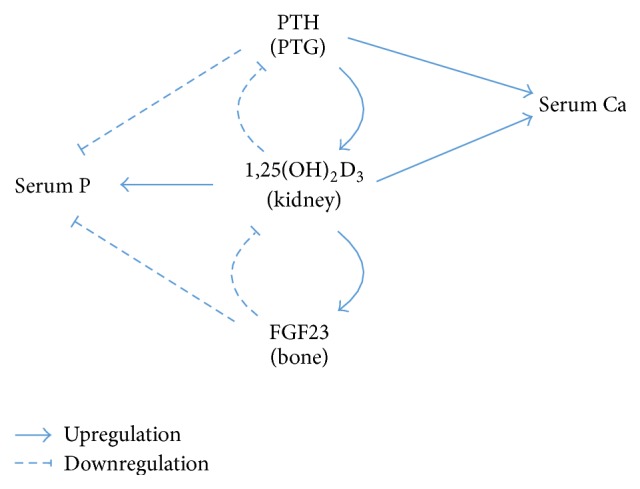
A simplified diagram showing the regulation of serum calcium and phosphate by parathyroid hormone, vitamin D, and FGF23. PTH: parathyroid hormone; PTG: parathyroid gland; Ca: calcium; P: phosphate.

Binding of FGF23 to Klotho/FGFR1c has two major physiological effects [19]. First, renal reabsorption of phosphate is inhibited, increasing phosphate excretion and decreasing serum phosphate levels. Second, FGF23 downregulates calcitriol production in the kidneys, while calcitriol upregulates the skeletal expression of FGF23. Thus, besides the well-known negative feedback loop of PTH and vitamin D, FGF23 and vitamin D form another negative feedback mechanism to provide precise regulation of serum calcium. In brief, FGF23 reduces vitamin D levels by regulating enzymatic conversion of 25(OH)D_3_ to 1,25(OH)_2_D_3_ (biologically active calcitriol) and 24,25(OH)_2_D_3_ (biologically inactive). Together, FGF23-Klotho, vitamin D, and PTH play key roles in maintaining calcium-phosphate homeostasis (Figure 1).

FGF23 and Klotho are also important in promoting development and slowing aging [20]. Klotho knockout mice and FGF23 knockout mice display a similar pattern of hyperphosphatemia and extensive vascular calcification [21–23]. In addition, Klotho knockout mice show blunted growth and premature aging [15, 24, 25]. Aging-associated phenotypes include osteoporosis [26], pulmonary emphysema, hypogonadism, skin atrophy [27], and premature death [25]. Recent studies have begun to suggest similar roles for Klotho in humans [28, 29]. For instance, children with organic growth hormone deficiency have considerably lower serum Klotho levels than normal children [30]. A large-scale study of 2734 older adults (aged 71–80 years) demonstrated a direct correlation between plasma Klotho concentrations and knee strength over time, suggesting the potential role of Klotho in modifying skeletal muscle strength [31].

These scientific findings are reminiscent of the TCM theory of “Kidneys Govern Bones.” The clinical manifestations of Kidney Essence Deficiency, including fatigue, delayed growth and development, pain and weakness in lower back and knees, and early aging [2], are strikingly similar to the phenotype of Klotho deficiency. Therefore, we propose that Klotho at least partly underlies the concept of Kidney Essence [32]. According to this theory, Kidney Essence is transformed into Kidney Qi, which at a molecular level corresponds to Klotho being transformed by FGFR1c into a high affinity FGF23 receptor, which binds to FGF23 to fulfill the function in regulating calcium-phosphate metabolism. In other words, the process of “Kidneys Govern Bones” is in part accomplished by kidney-expressed Klotho and bone-derived FGF23. Kidney Qi further generates Kidney Yang and Kidney Yin. 1,25(OH)_2_D_3_ resembles the function of Kidney Yang in promoting growth and bone strength, while biologically inactive 24,25(OH)_2_D_3_ resembles Kidney Yin in its role of limiting the production of 1,25(OH)_2_D_3_. Chinese medicine, in a simplified way, considers the intricate regulation of calcium-phosphate homeostasis as part of the Yin-Yang balance regulated by Kidney Essence.

Klotho is also present as a soluble form. Convincing studies demonstrate that the main source of soluble Klotho is the kidneys [33, 34]. Soluble Klotho levels in urine and blood are highly correlated with renal Klotho expression in rodents [33, 35]. Although Klotho can also be produced by the parathyroid gland, surgical removal of the parathyroid gland in humans with chronic kidney failure had no significant effect on their soluble Klotho levels [36]. As soluble Klotho is easily detectable and a decrease in its level has shown significant correlations in early phases of cardiovascular and kidney diseases [37–39], it has gained attention as a potential early diagnostic marker for these diseases.

## 3. Chronic Kidney Disease, Diabetic Mellitus, Metabolic Bone Disorder, and FGF23-Klotho 

Based on TCM theories, since kidneys govern bones we can deduce that any chronic condition with Kidney Essence Deficiency will likely lead to bone disorders. Indeed, recent scientific studies have provided convincing evidence to support this notion. Chronic kidney disease (CKD) and diabetes mellitus are two examples that demonstrate the relationship between Kidney Essence Deficiency and abnormal bone metabolism.

CKD, defined as gradual loss of renal function over time, is a global public health problem affecting 5–10% of the world population [40]. CKD progresses in 5 stages of severity, characterized by the levels of glomerular filtration rate (GFR), proteinuria, and pathological abnormalities of kidneys. Stage 5 CKD, also known as end-stage renal disease (ESRD), requires dialysis or kidney transplant. From a TCM perspective, Kidney Essence Deficiency is a main feature of CKD.

Diabetes mellitus has become an epidemic of modern times. In particular, type 2 diabetes mellitus (T2DM) affects 370 million people worldwide [41]. China was one of the first cultures to recognize diabetes [42]. TCM theory defines deficiency in Kidney Essence as a main cause of diabetes, through the inability to generate sufficient Kidney Qi to control glucose metabolism. This deficiency in Kidney Essence is further pronounced in diabetic nephropathy. Despite better control of blood glucose levels by medications, 42% of US patients with diabetes develop kidney disease [43]. Diabetic nephropathy is the most frequent cause of CKD in developed countries, accounting for 25–40% ESRD cases [44].

Metabolic bone disorder is common in CKD and diabetes. It can manifest in various forms, including abnormal calcium-phosphate metabolism, vascular calcification, and renal osteodystrophy.

Vascular calcification is a prevalent but covert presentation of bone disease, which in fact is a form of intravascular, extraskeletal, ectopic ossification. As an independent risk factor for morbidity and mortality, vascular calcification is particularly common in ESRD and T2DM [45] and contributes to microvascular and macrovascular complications of diabetes [46, 47]. Although it was once considered a passive process of calcium deposition on the vascular wall, a myriad of studies since the 1990s have clearly demonstrated that vascular calcification is an actively regulated pathophysiological process recapitulating chondroosteogenesis in atherosclerotic arteries and valves (reviewed in [45, 48]).

Klotho deficiency, as part of Kidney Essence Deficiency, is widely present in CKD and diabetes and may play an important role in the development of metabolic bone disorder. Many studies have demonstrated a concomitant decline in CKD patients of Klotho expression and renal function [49, 50]. Soluble Klotho levels are found to be positively correlated with GFR and negatively correlated with urine albumin levels. Lower serum Klotho levels are also often observed in diabetic subjects, and this is particularly obvious in the presence of diabetic nephropathy when renal function has deteriorated [51–53].

As discussed above, Klotho deficiency in the kidneys leads to reduced FGF23 mediated signaling. Therefore, the crosstalk between kidneys and bones breaks down. The mechanisms that regulate calcium-phosphate homeostasis and, in particular, that prevent hyperphosphatemia are disrupted. Consequently, calcium-phosphate imbalance develops, manifested as hyperphosphatemia and hypocalcaemia. On the one hand, this imbalance stimulates intravascular deposition of calcium and phosphate and osteochondrocytic differentiation, accelerating vascular calcification, which in turn aggravates microvascular and macrovascular complications of diabetes. On the other hand, the normal process of bone mineralization is disturbed, precipitating the development of the mineral and bone disorder of CKD (CKD-MBD) (reviewed in [54]).

**Figure 2:**
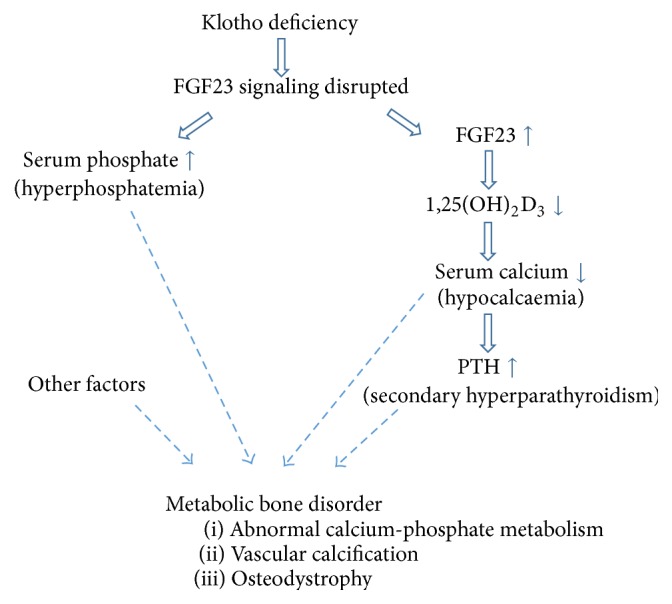
Consequences of disrupted FGF23-Klotho signaling. Keep in mind that this is a multifactorial multistep complex process.

The significance of Klotho and FGF23 partly lies in the possibility that their dysregulation represents the earliest alterations in CKD and diabetic nephropathy, prior to detectable changes in vitamin D and PTH and much earlier than the occurrence of hyperphosphatemia and hypocalcaemia [50]. Klotho deficiency, due to either reduced expression levels [28, 55] or gene polymorphisms that reduce FGF23-binding capacity [56–58], can lead to FGF23 resistance and compensatory increases in serum FGF23. This in turn contributes to a decrease in calcitriol and secondary elevation in PTH, further disrupting calcium-phosphate metabolism (Figure 2). Thus, Klotho deficiency is a key early step in kidney disease progression and a crucial factor in the development of diabetic complications [37, 59]. Therefore, Klotho is not only an early diagnostic marker, but also a promising therapeutic target for CKD and diabetes. In fact, the latest studies exploring Klotho upregulation in animal models of diabetes and diabetic nephropathy have shown promising results in protecting pancreatic islet cells and reducing renal fibrosis [60–63].

## 4. Chinese Herbal Medicine for Diabetic Nephropathy and CKD

As Kidney Essence Deficiency is a cause of diabetes as well as a consequence of diabetic nephropathy and CKD, improving renal functions by tonifying Kidney Essence with herbal medicine is an important TCM treatment strategy. Such herbal formulations have been widely used in Mainland China and Taiwan as an adjunct therapy for treating diabetes and CKD [64].

A recent survey [65] revealed that, during 1998–2008, 4% of the Taiwan population had T2DM, 13.9% of whom used Chinese herbal medicine to supplement conventional treatments. These T2DM patients had significantly lower risk for renal failure compared to those who did not use herbal medicine. Another study [66] tested Tianqi capsules, a Chinese herbal formulation containing 10 herb ingredients, in 420 subjects with impaired glucose tolerance in a double-blinded, randomized, placebo-controlled trial. At the end of the 12-month treatment, the percentage of subjects who had normal glucose tolerance was considerably higher in the Tianqi group (63.13%) versus placebo group (46.60%). Tianqi reduced the risk of developing T2DM by 32.1%. No toxicity or severe adverse effects were observed. This study demonstrated the safety of Tianqi and its potential for preventing diabetes in subjects with impaired glucose tolerance.

Our clinical studies have also demonstrated the therapeutic value of Chinese herbal medicine for treating and preventing CKD. One example is Shen-An extracts, a kidney-tonifying toxin-clearing herbal formulation, which as we have shown improves renal function and clinical symptoms in patients with chronic renal failure [67].

According to TCM [68], Kidney Essence Deficiency in diabetic nephropathy and CKD leads to inability of the kidneys to retain essential substances (e.g., albumin and red blood cells) and to clear turbidity (i.e., metabolic waste products). As a result, proteinuria and hematuria occur, and high levels of waste products accumulate in the blood. This notion is similar to the understanding of CKD pathogenesis in Western medicine. Therefore, the TCM strategy for treating CKD is to tonify kidneys and drain turbidity, in other words, to improve renal function and facilitate clearance of waste products. The formulation of Shen-An reflects this concept.

Shen-An extracts comprise three ingredients, Yin Yang Huo (*Herba Epimedii*), Huang Qi (*Astragalus membranaceus*), and Da Huang (*Rheum officinale*, also known as Chinese rhubarb).* Herba Epimedii* acts as the chief herb,* Astragalus membranaceus* acts as the deputy herb, and* Rheum officinale* acts as the assistant and envoy herb in their hierarchy of importance.* Herba Epimedii*,* Astragalus membranaceus*, and* Rheum officinale* are mixed in the ratio of 2 : 2 : 1 by the weight of dry herbs. Semibionic extraction (SBE) process was used to maximize the extraction rate of the chief and deputy herbs [69]. The optimal extraction condition was 5-hour water extraction containing 3 consecutive steps, that is, pH2 extraction for 2 hours, pH7 extraction for 2 hours, and pH9 extraction for 1 hour. The extracted rates of* Herba Epimedii* and* Astragalus membranaceus* were 95.95% and 95.62%, respectively.

*Herba Epimedii* is an important herb in TCM practice for tonifying Kidney Yang and strengthening bones. Icariin, the main bioactive compound in* Herba Epimedii*, has numerous therapeutic activities that are osteoprotective, neuroprotective, immunoprotective, and cardioprotective and that support reproductive functions [70]. Protective effects have also been demonstrated in experimental diabetic nephropathy [71, 72]. Likewise,* Astragalus membranaceus* contains a wide range of constituents with biological activities [73]. Among them, astragalus saponin IV (also known as astragaloside IV) can ameliorate proteinuria and renal fibrosis in rodent models of diabetic nephropathy [74, 75]. The traditional use of* Rheum officinale* is to drain turbidity and detoxify the body. Recent studies have identified its nephroprotective mechanisms, mainly by promoting excretion of uremic toxin through the colon and attenuating renal interstitial fibrosis [76, 77]. Altogether, these three ingredients improve renal function and clear waste products.

We carried out a series of experiments to understand the therapeutic mechanisms of Shen-An in diabetic nephropathy and CKD. First, we investigated Shen-An in a mouse model of streptozotocin-induced diabetic nephropathy, comparing three doses of Shen-An and irbesartan (an angiotensin II receptor antagonist) [78]. Following gavage administration for 4 weeks, Shen-An extracts had no effect on blood glucose but significantly reduced urine albumin, serum creatinine (SCr), and glomerular sclerosis index. The most advantageous result was observed with the higher dosage of Shen-An, which was comparable to that of irbesartan. Further experiments showed that the nephroprotective effect of Shen-An was correlated to its suppression of transcription activator protein 1 (AP-1), transforming growth factor *β*1 (TGF-*β*1) [78], and advanced glycation end-product (AGE) expression [79] in the kidneys.

We then investigated the effect of Shen-An in a rat model of renal osteodystrophy induced by 5/6 nephrectomy and high phosphorus water intake [80, 81]. One month following 5/6 nephrectomy, remnant kidney rats on high phosphorus water began to develop renal failure (evidenced by marked elevations in blood urea nitrogen (BUN) and SCr and significant decline in creatinine clearance rate (CCR) and hemoglobin), hyperphosphatemia, hypocalcaemia, and hyperparathyroidism, all of which continued to worsen during the period of the study. These rats also showed extensive histopathological changes in the remnant kidney and developed osteodystrophy, with a loss in bone mineral density (BMD). As expected, there was a significant decrease in renal Klotho expression and increase in skeletal FGF23 expression. Two doses of Shen-An and one dose of calcitriol were started one month after 5/6 nephrectomy and continued for 8 weeks. At the end of the study, Shen-An partially corrected the abnormal changes in serum calcium, phosphate, and alkaline phosphatase levels, reduced pathological deterioration of the kidney, and significantly improved renal functions (BUN, SCr, and CCR), anemia (hemoglobin), and bone density (BMD). These improvements were accompanied by partially restored renal Klotho expression and inhibition of abnormally high FGF23 production. It is worth noting that both doses of Shen-An provided greater protection than calcitriol, and the low dose of Shen-An was better than the high dose.

Thus, our research has demonstrated that Shen-An extracts can effectively delay the progression of diabetic nephropathy and improve calcium-phosphate metabolism and kidney functions in renal osteodystrophy. Its effect is comparable to or better than that of irbesartan and calcitriol. The mechanisms underlying its protective impact may involve upregulation of renal Klotho expression, downregulation of skeletal FGF23 expression, and reduction of AP-1, TGF-*β*1, and AGE in the kidneys.

## 5. Summary

Vitamin D and FGF23-Klotho signaling pathways have provided important insight into the scientific basis of the TCM theory “Kidneys Govern Bones” and have helped us understand how vascular complications and metabolic bone disorder evolve in CKD and diabetes mellitus. Chinese herbal medicine, such as Shen-An extracts, appears to exert its therapeutic effects in treating diabetic nephropathy and renal osteodystrophy at least partly through these mechanisms.

It is important to point out that with improved awareness and diagnosis of diabetes, as well as wide use of glucose-lowering medications, the majority of diabetic patients have well-managed blood glucose levels. The typical symptoms, for example, polyuria, polydipsia, and polyphagia, are no longer prevalent. However, despite such progress, microvascular and macrovascular complications continue to develop and have become the leading causes of morbidity and mortality in diabetics. Likewise, the current status of CKD treatment is not encouraging. There is no cure for CKD. Current strategies relying on blockade of renin-angiotensin system (RAS) can only delay the onset of ESRD and often cause serious side effects. Therefore, there is an urgent need for better treatments for both diseases.

It is our hope that a renewed study of TCM theories, herbal formulations, and ingredients will inspire new ideas and therapies for diabetes and CKD treatment. Not only can integrative medicine improve blood glucose levels and kidney function, but we also believe that astutely combining Eastern and Western concepts holds great promise for facilitating discovery of novel approaches that effectively prevent and treat long-term complications of diabetes and CKD.
